# Three-layer heterogeneous mammographic phantoms for Monte Carlo simulation of normalized glandular dose coefficients in mammography

**DOI:** 10.1038/s41598-020-59317-4

**Published:** 2020-02-10

**Authors:** Tien-Yu Chang, Kuan-Jen Lai, Chun-Yuan Tu, Jay Wu

**Affiliations:** 1 Department of Radiology, Cheng Hsin General Hospital, Taipei, Taiwan; 2 Department of Biomedical Imaging and Radiological Sciences, National Yang-Ming University, Taipei, Taiwan; 3 Department of Radiology, Mackay Memorial Hospital, Taipei, Taiwan

**Keywords:** Health care, Medical imaging, Risk factors

## Abstract

Normalized glandular dose (DgN) coefficients obtained using homogeneous breast phantoms are commonly used in breast dosimetry for mammography. However, glandular tissue is heterogeneously distributed in the breast. This study aimed to construct three-layer heterogeneous mammographic phantoms (THEPs) to examine the effect of glandular distribution on DgN coefficient. Each layer of THEPs was set to 25%, 50%, or 75% glandular fraction to emulate heterogeneous glandular distribution. Monte Carlo simulation was performed to attain mean glandular dose (MGD) and air kerma at 22–36 kVp and W/Al, W/Rh, and W/Ag target–filter combinations. The heterogeneous DgN coefficient was calculated as functions of the mean glandular fraction (MGF), breast thickness, tube voltage, and half-value layer. At 50% MGF, the heterogeneous DgN coefficients for W/Al, W/Rh, and W/Ag differed by 40.3%, 36.7%, and 31.2%. At 9-cm breast thickness, the DgN values of superior and inferior glandular distributions were 25.4% higher and 29.2% lower than those of uniform distribution. The proposed THEPs can be integrated with conventional breast dosimetry to consider the heterogeneous glandular distribution in clinical practice.

## Introduction

Breast cancer is the second most common cause of cancer deaths among women^1^, and its incidence increases annually. X-ray mammography has become a primary tool for breast cancer screening because of the high sensitivity and specificity for microcalcification and mass detection^2^. Mammography is also adopted for the high effectiveness/cost ratio. However, the glandular tissue in the breast is sensitive to radiation. The radiation exposure during mammography may increase the risk of radiation-induced secondary breast malignancy^3^. Therefore, assessment of glandular dose in the breast is crucial.

In modern breast dosimetry, one of the most critical parameters is the normalized glandular dose (DgN) coefficient, which is obtained using Monte Carlo simulation of mean glandular dose (MGD) in a breast phantom^4^. Boone used simple homogeneous breast phantoms to simulate the breast absorbed dose and applied a *G* factor to calculate MGD^5^. He further proposed monoenergetic DgN coefficients to facilitate rapid calculation of the DgN coefficient for arbitrary X-ray energy spectra^6^. Dance *et al*.^7^ employed homogeneous semicylindrical phantoms to assess MGD conversion factor *g*, breast composition factor *c*, and X-ray spectrum factor *s*. The authors further supplemented the conversion factors for the high-energy spectra used for contrast enhanced digital mammography by using the same homogeneous breast model^8^. The above method is now the standard breast dosimetry protocols for mammography in the United Kingdom, European Union, and IAEA and is also adopted in the quality assurance protocols in USA. Sarno *et al*.^9^ used homogeneous semi-cylinder and ad hoc shaped phantoms to exam MGD in mammography and calculate the t-factor (relative glandular dose, RGD) and T-factor in digital breast tomosynthesis (DBT).

Three-dimensional (3D) imaging modalities, including computed tomography (CT)^10,11^, breast CT (bCT)^12,13^, and magnetic resonance imaging (MRI)^14^, have been used to construct high-resolution breast voxel phantoms to examine the effect of glandular distribution on DgN coefficient. The results revealed 24% to 43% differences in the DgN values between voxel phantoms and homogeneous phantoms^10,12^. Wang *et al*.^15^ used breast tissue information adopted from Chinese females to build a series of 3D detailed breast models, which include the retromammary fat, laciferous duct and lobule, intraglandular fat, glandular tissue, subcutaneous fat, Cooper’s ligament, and skin. The DgN values of the 3D models were 5.4%–38.0% lower than those of the homogeneous model. Sarno *et al*.^16^ compared four types of homogeneous breast models with 20 patient-specific digital breast phantoms. The MGD differences were varied from 43% to −28%, inferring the importance of glandular distribution in the breast models.

The heterogeneous breast phantoms are usually constructed based on 3D medical images of specific subjects, who cannot represent the entire population. In addition, complete heterogeneous DgN data sets for various tube voltages, target–filter combinations, beam qualities, breast thicknesses, and glandular distributions are still scarce for MGD and diagnostic reference level (DRL) assessment^17^. This study proposed digital three-layer heterogeneous mammographic phantoms (THEPs) and used the Monte Carlo method to obtain DgN coefficients for different target–filter combinations in mammography. THEPs possess the characteristic of heterogeneous glandular distribution, and the corresponding DgN coefficients can be integrated into the existing breast dosimetric systems.

## Materials and Methods

### Monte carlo simulation

The geometric models of mammography and THEPs were constructed using Monte Carlo N-Particles (MCNP) transport code (version 6.1). Particle tracking was performed using the photon mode, including the following interactions: coherent scattering, photoelectric effect, and Compton scattering. The photon energy cutoff was set to 5 keV. Due to the short range of secondary electrons produced in mammography, the kinetic energy of electrons was considered to be locally deposited^18^. Fifty million photons were simulated in each case to reduce the coefficient of variation to less than 2%.

### THEP modelling

**Figure 1:**
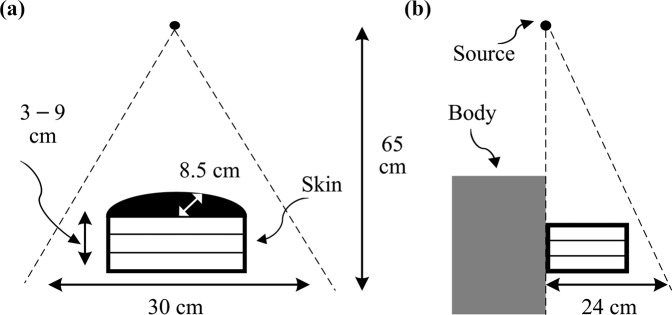
(**a**) Three-layer heterogeneous mammographic phantom in the form of a semicylinder with a radius of 8.5 cm and an external skin layer of 0.4 cm, and (**b**) the geometry of mammography with a THEP and a torso phantom.

The THEP was a semicylinder with a radius of 8.5 cm, thicknesses of 3–9 cm, and a skin layer of 0.4 cm (Fig. 1). It was constructed to emulate the compressed breast in mammography. The inner compartment of THEP was evenly divided into three layers; the glandular fraction (GF) in each layer can be set to 25%, 50%, or 75%. When the GF of all three layers is identical, the THEP is consistent with the phantom used in conventional breast dosimetry of mammography^6^ and is referred to as the three-layer homogeneous mammographic phantom (THOP).

Heterogeneous glandular distributions were simulated through the GF combinations of the three layers. For example, at 50% mean glandular fraction (MGF), the following distributions are possible: uniform distribution (50%:50%:50%), concentrated distribution (25%:75%:50%), inferior distribution (25%:50%:75%), and superior distribution (75%:50%:25%). The elemental composition and density of the tissue with 25%, 50%, and 75% GF as well as the skin were derived from the information of the glandular tissue, adipose tissue, and skin proposed by Hammerstein *et al*.^19^ (Table 1).

**Table 1:** Elemental composition and density of the tissue with 25%, 50%, and 75% GF as well as the skin.

Tissue	Density (g/cm^3^)	Composition (weight percent, %)			
		H	C	N	O
25% GF tissue	0.955	11.0	51.0	2.1	35.7
50% GF tissue	0.982	10.7	40.1	2.5	46.4
75% GF tissue	1.010	10.5	29.3	2.9	57.0
Skin	1.090	9.8	17.8	5.0	66.7

### Mammography modelling

The focal spot of the X-ray tube in mammography was set as an isotropic point source, which was 65 cm away from an image receptor. The THEP was placed at 1.2 cm above the image receptor, and a 30 × 20 × 40 cm^3^ torso phantom was placed behind the THEP (Fig. 1b). The material of the torso phantom was taken from the soft tissue in the ICRU-44 report^20^. The compression paddle and support plate were not simulated. The source term was generated by the Tungsten Anode Spectral Model using Interpolating Polynomials (TASMIP) software to produce 22–36 kVp tungsten spectra with rhodium, silver, and aluminium filters, respectively^21^. The half-value layers (HVLs) of the energy spectra for commonly used tube voltages are displayed in Table 2.

**Table 2:** Half-value layers of the energy spectra for commonly used exposure parameters in mammography.

Tube voltage (kVp)	Target–filter combinations	Filter thickness (μm)	HVL (mm Al)
26	W/Rh	50, 65, 80, 95	0.460, 0.524, 0.578, 0.622
26	W/Ag	50, 65, 80, 95	0.471, 0.553, 0.614, 0.667
26	W/Al	500, 650, 750, 850	0.302, 0.377, 0.423, 0.465
30	W/Rh	50, 65, 80, 95	0.499, 0.563, 0.613, 0.655
30	W/Ag	50, 65, 80, 95	0.541, 0.620, 0.684, 0.740
30	W/Al	500, 650, 750, 850	0.356, 0.440, 0.499, 0.549
36	W/Rh	50, 65, 80, 95	0.548, 0.608, 0.652, 0.690
36	W/Ag	50, 65, 80, 95	0.606, 0.679, 0.740, 0.792
36	W/Al	500, 650, 750, 850	0.434, 0.528, 0.600, 0.661

### MGD and air kerma simulation

Photons were simulated from the focal spot to the image receptor, and the energy deposited in each layer of the THEP was recorded. The MGD was calculated as follows:

1 $$MGD=\frac{{\sum }_{i}{G}_{i}(E)\times {E}_{i}}{{\sum }_{i}V\times {\rho }_{i}\times {f}_{{\rm{g}},i}}$$

2 $$G(E)=\frac{{f}_{{\rm{g}}}\times {(\frac{{\mu }_{{\rm{en}}}}{\rho })}_{{\rm{g}}}}{{f}_{{\rm{g}}}\times {(\frac{{\mu }_{{\rm{en}}}}{\rho })}_{{\rm{g}}}+(1-{f}_{{\rm{g}}})\times {(\frac{{\mu }_{{\rm{en}}}}{\rho })}_{{\rm{a}}}}$$

where *E*_*i*_ is the energy deposited in layer *i*, *V* is the volume of each layer, and *ρ*_*i*_ is the tissue density in layer *i*. *G*(*E*) is the energy absorption ratio of the glandular tissue to total breast calculated by the mass energy absorption coefficients of glandular tissue (*μ*_en_/*ρ*)_g_ and adipose tissue ${({\mu }_{en}/\rho )}_{a}$; *f*_*g*_ and (1 − *f*_*g*_) are the weight percentages of glandular and adipose tissues, respectively. In addition, we constructed the geometric model of a pancake ionisation chamber with a radius of 14.5 mm and thickness of 2 mm to simulate air kerma in the active volume. The chamber was filled with dry air and placed at the position of the THEP. The breast and torso phantoms were removed to avoid backscattered photons. The compression paddle was not simulated as well to avoid large angular spread of X-ray photons on the detector surface^22^.

### DgN calculation

Twenty-seven glandular distributions were generated from different combinations of GF in each layer of the THEP. The DgN coefficient was calculated by dividing the MGD by the air kerma for different X-ray energy spectra and 3–9 cm breast thicknesses, as follows:

3 $$DgN=\frac{MGD}{K}$$

where *K* is the air kerma. For verifying the Monte Carlo modelling, THOPs with 25%, 50%, and 75% MGF were employed to attain the DgN coefficients at the W/Ag target–filter combination for breast thicknesses of 3, 5, and 7 cm and tube voltages of 26, 28, 30, 32, and 34 kVp. The results were compared with those obtained by Nosratieh *et al*.^23^. Subsequently, THEPs were used to obtain the heterogeneous DgN coefficients for concentrated, inferior, and superior glandular distributions. The relationships between the heterogeneous DgN coefficient, MGF, breast thickness, tube voltage, and HVL were established.

## Results

### Validation of Monte Carlo code

We used MCNP 6.1 with the MCPLIB84 cross-section library to simulate the case 3 in the AAPM TG-195 report for mammography dosimetry^24^. The breast model was a semicylinder with a radius of 98 mm, thicknesses of 46 mm, and skin layer of 2 mm. The interior composition of the model was a homogeneous mixture of 80% adipose tissue and 20% glandular tissue. A polymethyl-methacrylate rectangular box with a dimension of 140 × 260 × 2 mm^3^ was added above and below the breast model as compression and support paddles. The distance between the source and compression paddle was 59.3 cm, and photons with 16.8 keV were emitted from the source isotropically. The result of breast energy deposition per photon was 4707 eV/photon (CV = 0.42%). Compared with the result of TG-195, the error is less than 0.2%, indicating the credibility of the Monte Carlo code.

### Verification of DgN coefficients

**Figure 2:**
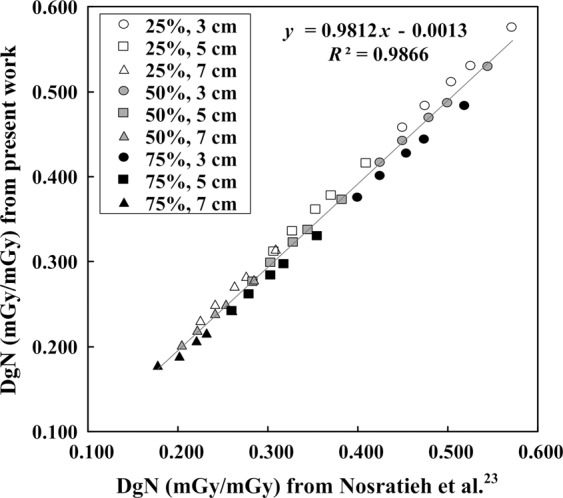
Scatter plot of the DgN coefficients of THOPs and those obtained by Nosratieh *et al*.^23^. The linear regression result showed a good linearity with an *R*^2^ of 0.9866.

Figure 2 compares the DgN coefficients of THOPs and those obtained by Nosratieh *et al*.^23^. The linear regression result revealed a favorable linearity (*y = *0.9812*x* − 0.0013, *R*^2^ = 0.9866) with no significant differences between the two sets of DgN values (*p* = 0.727). The mean DgN differences at 25% and 50% MGF were 2.2% and 1.8%, respectively, whereas the difference slightly increased to 6.4% at 75% MGF. By using the same X-ray spectral model proposed by Boone *et al*.^21^, Sarno *et al*.^25^ also compared their simulation data with those proposed by Nosratieh *et al*., and showed that 5% higher DgN coefficients on average. This result infers that the DgN coefficients of THOPs may be approximately 7% lower that the results obtained by Sarno *et al*.

### Heterogeneous DgN coefficient and mean glandular fraction

**Figure 3:**
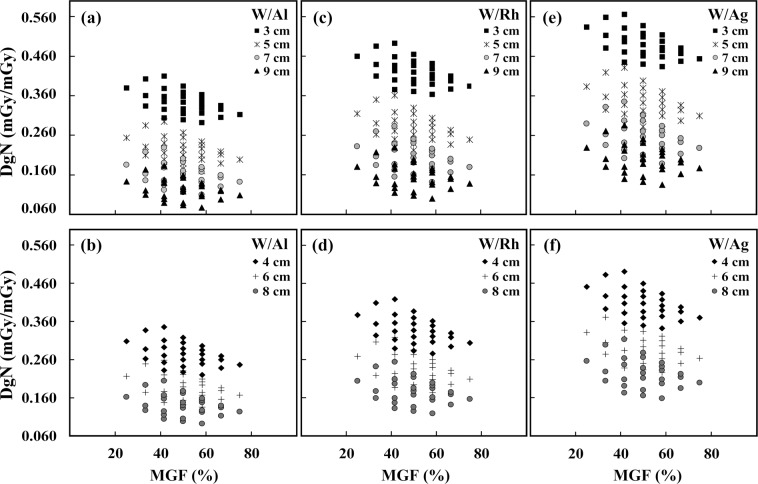
Heterogeneous DgN coefficient versus mean glandular fraction for three commonly used exposure conditions: (**a**)(**b**) 26 kVp, Al filter, and 0.423-mm HVL, (**c**)(**d**) 30 kVp, Rh filter, and 0.563-mm HVL, and (**e**)(**f**) 36 kVp, Ag filter, and 0.679-mm HVL. Upper row and lower row represent breast thicknesses of 3, 5, 7, and 9 cm and breast thicknesses of 4, 6, and 8 cm, respectively.

Figure 3 illustrates the heterogeneous DgN coefficients of THEPs as a function of MGF for three commonly used exposure conditions, (1) 26 kVp, Al filter, and 0.423-mm HVL, (2) 30 kVp, Rh filter, and 0.563-mm HVL, and (3) 36 kVp, Ag filter, and 0.679-mm HVL. The DgN coefficient had an inverse trend with MGF and breast thickness. The scattered data points for a given breast thickness and MGF represent the heterogeneous DgN coefficients resulting from different glandular distributions. At 4-cm breast thickness and 50% MGF, the differences in the heterogeneous DgN coefficients for the W/Al, W/Rh, and W/Ag target–filter combinations were 40.3%, 36.7%, and 31.2%, respectively.

### Heterogeneous DgN coefficient and breast thickness

**Figure 4:**
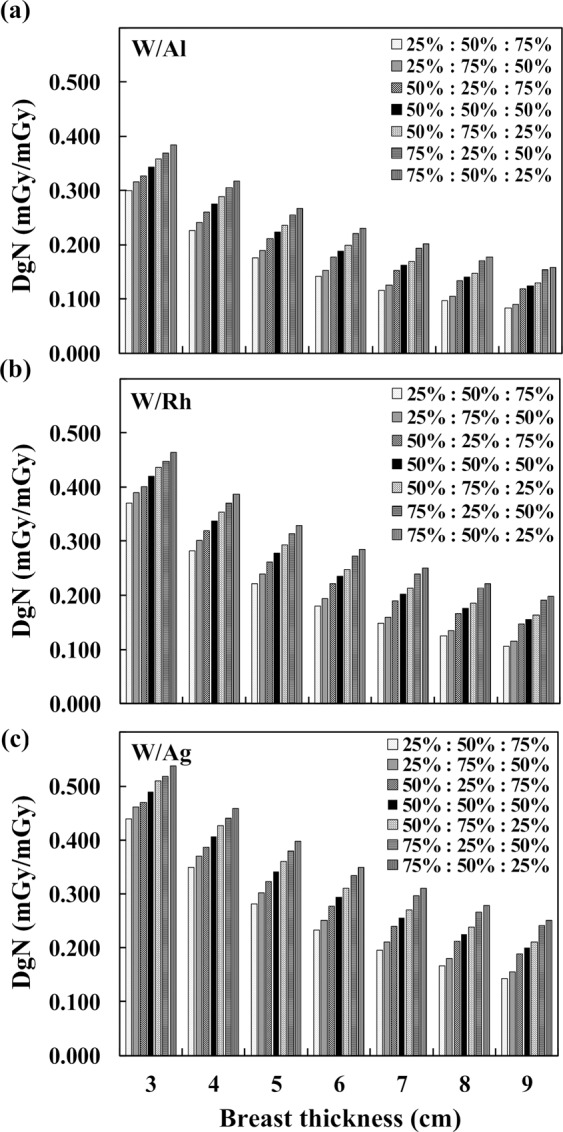
Heterogeneous DgN coefficient versus breast thickness for the three commonly used exposure parameters of (**a**) 26 kVp, W/Al, and 0.423-mm HVL, (**b**) 30 kVp, W/Rh, and 0.563-mm HVL, and (**c**) 36 kVp, W/Ag, and 0.679-mm HVL.

Figure 4 depicts the heterogeneous DgN coefficients of THEPs for breast thicknesses of 3 to 9 cm for the three commonly used exposure conditions at 50% MGF. The DgN coefficient decreased with an increase in breast thickness. In the situation of superior glandular distribution, the DgN value was higher than that of uniform distribution; conversely, the DgN value of inferior distribution was lower than that of uniform distribution. At 4-cm breast thickness, the DgN coefficient of the upper concentrated distribution (50%:75%:25%) was higher than that of the lower concentrated distribution (25%:75%:50%) by 19.3%, 17.6%, and 15.1% for W/Al, W/Rh, and W/Ag target–filter combinations.

### Heterogeneous DgN coefficient and tube voltage

**Figure 5:**
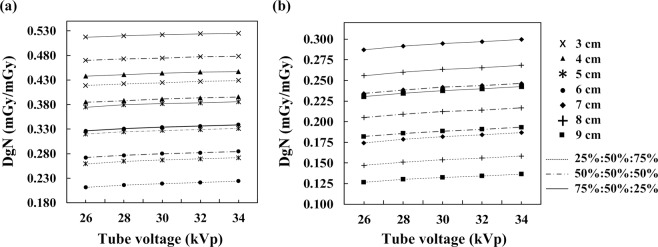
Heterogeneous DgN coefficient versus tube voltage for (**a**) 3 to 6-cm small breasts and (**b**) 7 to 9-cm large breasts at W/Ag target–filter combination, 0.658-mm HVL, and 50% MGF. The DgN coefficient was positively correlated with tube voltage.

Figure 5 shows the heterogeneous DgN coefficients of THEPs as a function of tube voltage at W/Ag target–filter combination, 0.658-mm HVL, and 50% MGF. The DgN coefficient was positively correlated to tube voltage but negatively correlated to breast thickness. At 26 kVp, the mean DgN coefficient of inferior glandular distribution of small breasts (3–6 cm) was 16.8% lower than that of uniform distribution, whereas the mean DgN value of superior distribution was 15.3% higher than that of uniform distribution. When the tube voltage increased to 34 kVp, the mean DgN coefficients of inferior and superior distributions were 15.9% lower and 14.6% higher than that of uniform distribution, respectively. The results suggest that the effect of glandular distribution on DgN coefficient slightly decreases with increasing tube voltage.

### Heterogeneous DgN coefficient and HVL

**Figure 6:**
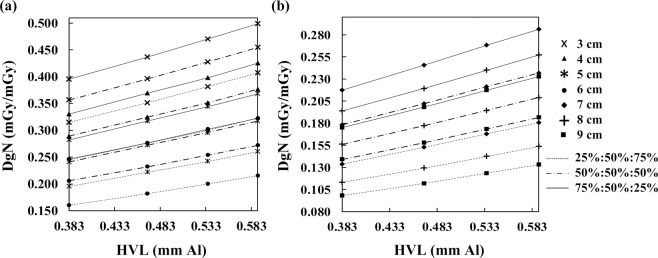
Heterogeneous DgN coefficient versus HVL for (**a**) 3 to 6-cm small breasts and (**b**) 7 to 9-cm large breasts at 32 kVp, W/Al target–filter combination, and 50% MGF. The DgN coefficient was positively correlated with HVL.

Figure 6 shows the heterogeneous DgN coefficients of THEPs for various HVLs at 32 kVp, W/Al target–filter combination, and 50% MGF. The DgN value was positively correlated with HVL. At 0.469-mm HVL and 4-cm breast thickness, the DgN coefficient of inferior glandular distribution was 15.0% lower than that of uniform distribution, whereas the DgN coefficient of superior distribution was 13.9% higher than that of uniform distribution. At the same HVL but 9-cm breast thickness, the DgN coefficients of inferior and superior distributions were 29.2% lower and 25.4% higher than that of uniform distribution. The glandular distribution has a stronger impact on DgN coefficient in larger breasts.

### Heterogeneous DgN coefficient for clinical exposure conditions

Table 3 lists clinical exposure parameters and heterogeneous DgN coefficients for different glandular distributions of small, medium, and large breasts at 50% MGF. At the breast thicknesses of 4, 6, and 8 cm, the DgN coefficients of superior distribution were 15.6%, 21.3%, and 23.0% higher than that of uniform distribution, respectively; in contrast, the DgN values of inferior distribution were 17.8%, 23.8%, and 26.5% lower than that of uniform distribution. The results again reveal that breast thickness has a strong influence of glandular distribution on DgN coefficient.

**Table 3:** Clinical exposure parameters and heterogeneous DgN coefficients for superior, inferior, and uniform distributions of small, medium, and large breasts.

Tube voltage (kVp)	Target–filter combination	HVL (mm Al)	CBT (cm)	DgN (mGy/mGy)		
				superior	uniform	inferior
26	W/Al	0.423	4	0.318	0.275	0.226
30	W/Rh	0.563	6	0.285	0.235	0.179
36	W/Ag	0.679	8	0.278	0.226	0.166

## Discussion

This study showed that the DgN coefficient is negatively correlated with breast thickness, which is consistent with the earlier investigation by Wu *et al*.^26^ and Santos *et al*.^27^. The primary reason for this trend is that the breast mass increases with increasing breast thickness, thus lowering the MGD and the corresponding DgN coefficient. The DgN coefficient also has an inverse relationship with MGF, which is mainly because the tissue with higher GF attenuates more X-ray photons and lowers the percent depth dose.

At 50% MGF, seven glandular distributions can be generated in THEPs, mainly uniform (50%:50%:50%), inferior (25%:50%:75%), superior (75%:50%:25%), lower concentrated (25%:75%:50%), and upper concentrated (50%:75%: 25%) distributions. The remaining two, 50%:25%:75% and 75%:25%:50%, are relatively uncommon in clinical practice. It is worth mentioning that the uniform distribution is the one used in the conventional mammographic dosimetry. The glandular distribution affects the DgN coefficient substantially. When the GF of the first layer of THEPs increases, the DgN value increases. This trend also applies to the upper concentrated glandular distribution, which has a higher DgN value than the lower concentrated distribution. These results can be explained by the exponential attenuation of X-ray photons that deposit most of their energy near the photon entrance side of the breast. Higher GF in the first layer thus substantially contributes to the MGD. In clinical practice, inferior and lower concentrated glandular distributions are more common than other types of distributions, indicating the use of homogeneous DgN coefficient may result in overestimation of MGD.

Higher tube voltage and HVL are often used for large breasts in mammography. As tube voltage increases, more energy is deposited in the breast regardless of the glandular distribution, resulting in a higher MGD and DgN coefficient. However, at high kVp, the effect of glandular distribution on DgN coefficient slightly decreases because the difference in the mass energy absorption coefficient between adipose and glandular tissues decreases. For reducing skin dose, additional filters are added to attenuate low-energy photons and consequently elevate the HVL of X-ray spectra. Similarly, the DgN coefficient increases and the effect of glandular distribution on DgN coefficient decreases because of the increase in the average energy of X-ray.

Hernandez *et al*.^12^ used bCT images to construct heterogeneous phantoms, indicating the DgN coefficient can vary by −22.1% to 28.5%. The results of the present study revealed a similar trend. Additionally, we showed that glandular distribution has a stronger effect on DgN coefficient for larger breasts. The DgN value varies from −29.2% to 25.4% at 9-cm breast thickness, whereas the DgN differences are from −15.0% to 13.9% in medium-sized breasts. These findings conclude that using the DgN coefficient obtained from simple homogeneous phantoms may cause a 30% error in MGD evaluation.

In clinical practice, the MGF can be determined using Breast Imaging Reporting and Data System (BI-RADS) or other clinically available software through mammogram analysis, such as the Laboratory for Individualized Breast Radiodensity Assessment (LIBRA)^28^ and Volpara^29^. The glandular distribution pattern can be evaluated from the mediolateral view of mammograms by drawing regions of interest (ROIs) in the upper, middle, and lower regions inside the breast, respective. The grayscale ratio between the ROIs is used as the basis for breast distribution. Other 3D imaging modalities, such as DBT, bCT, and MRI, are also suitable for estimating the GF ratio in each layer of THEPs. By using the threshold method, the GF in each layer can be calculated as the number of glandular pixels divided by the number of whole breast pixels in that layer.

There are various sources of systematic errors in the calculation of DgN coefficients. Firstly, in the elemental composition and density of materials, several data sources, including Hammerstein’s^19^ and ICRU 44^20^, show a wide variability for both adipose and glandular tissues. Chen *et al*.^30^ compared the linear attenuation coefficients of adipose and glandular tissues taken from different studies and showed that the results of glandular tissue from the Hammerstein’s data are in good agreement with those from the other three studies^30–32^. However, at lower energies, the linear attenuation coefficients of adipose tissue are slightly inconsistent among the three studies, which inevitably increases the uncertainty of MGD simulation. To further investigate this uncertainty, we used the data from ICRU 44 instead of Hammerstein’s data in THOPs, and the results shows approximately 3% uncertainty in MGD. Secondly, oversimplified breast models have been used for different clinical breast dose assessment protocols, which overestimate MGD by about 30%^12^. If the purpose of MGD evaluation is to reflect the average population breast dose for radiation protection and DRL assessment, mildly conservative overestimation suits the basic principles of radiation protection^33^. Finally, the systematic errors may come from the Monte Carlo code and its cross-section library. Our simulation result of the case 3 in the AAPM TG-195 report for mammography dosimetry has a difference less than 0.2% compared with the result of TG-195. Different Monte Carlo programs, including EGSnrc, Geant4, MCNPX, and Penelope, may also cause 0.2% uncertainty in MGD evaluation^24^.

The purpose of using heterogeneous phantoms derived from clinical CT scans for MGD simulations is to overcome the problem of non-uniform distribution of breast tissue. New clinical breast dosimetric systems should be developed from this perspective. However, existing clinical systems still use simple homogenous phantoms in their glandular dose assessment^34^: Philips uses Dance’s system, GE uses Wu’s system, and Hologic uses Boone’s system. Our proposed THEPs are compatible with the simple phantom used in clinical dosimetric systems and are intended to correct the problem of overestimated MGD originating from homogeneous breast tissue assumption. For example, the glandular tissue in breasts is often inferiorly distributed. By using the THEP protocol, the dose can be reduced by 17% to 26% according to the results listed in Table 3. Before the DgN coefficients obtained from those heterogeneous and voxel phantoms find a widespread use in digital mammography systems, correction of the DgN results can be obtained with the help of THEP.

The thickness of the skin layer has a crucial influence on MGD assessment. Dance^35^ and Boone^5^ employed the breast models with 5- and 4-mm-thick skin layers in the currently used dosimetric systems, respectively. However, by using bCT images, Huang *et al*.^36^ and Vedantham *et al*.^37^ analysed the breast skin thickness to be 1.45 mm. The thick skin layer attenuates more X-ray photons, thereby reducing the MGD by up to 27%^18,38^. To be compatible with these clinically used breast dosimetric systems, the skin thickness of THEPs was set to 4 mm. Although the calculated DgN coefficients may indeed be underestimated, the ratio of DgN of different glandular distributions to DgN of uniform glandular distribution remains valid. This ratio can be fed into other breast dose assessment systems to correct for the skin thickness problem, while solving the problem of non-uniform distribution of breast tissue at the same time. Finally, the question of optimising the skin thickness of THEPs merits further research.

## Conclusion

This study employed THEPs to investigate the DgN coefficient for uniform, concentrated, superior, and inferior glandular distributions. The results indicate that DgN coefficient is negatively correlated to breast thickness and MGF, whereas it is positively correlated to tube voltage and HVL. The effect of heterogeneous glandular distribution on DgN coefficient is significantly stronger for large breasts, but it is slightly weaker at high photon energy and HVL. The glandular distribution may result in a 29.2% difference in the DgN coefficient at 50% MGF. The proposed THEPs and heterogeneous DgN coefficients can consider the breast thickness, mean glandular fraction, glandular distribution, tube voltage, beam quality, and target–filter combination, while facilitating the integration with conventional breast dosimetry to provide clinical MGD and DRL evaluation.

## Data Availability

The datasets generated and analysed during the current study are available from the corresponding author on reasonable request.
